# Validation of house-keeping genes for normalization of gene expression data during diurnal/circadian studies in rice by RT-qPCR

**DOI:** 10.1038/s41598-018-21374-1

**Published:** 2018-02-16

**Authors:** Nitin Jain, Satyam Vergish, Jitendra P. Khurana

**Affiliations:** 1 Department of Plant Molecular Biology, University of Delhi South Campus, New Delhi, 110021 India; 2 Interdisciplinary Centre for Plant Genomics, University of Delhi South Campus, New Delhi, 110021 India

## Abstract

The circadian clock in plants is the intrinsic rhythmic expression of thousands of genes in a 24 h period, which is set by the day-night cycles in the environment. The study of the circadian clock often requires expression profiling of genes over a large number of samples for which RT-qPCR is invariably used. Reliability of the results depends largely on the house-keeping genes, which serve as control and thus should be chosen carefully to prevent erroneous results. In this study, ten house-keeping genes were chosen from rice for stability analysis with 48 tissue samples harvested from plants subjected to diurnal/circadian cycles. Although, all the genes were found to be stable, however, six of them showed cyclic expression patterns and caused major changes in the expression profiles of the target genes when used to normalize their expression data, thereby making them poor candidates for diurnal/circadian studies. In conclusion, reference genes need to be selected for diurnal/circadian studies with extra caution as more than 80% of transcriptome in plants undergoes cycling, which remains undetected by the gene stability assessment software and can severely affect the RT-qPCR based gene expression profiling. The geometric mean of two or more most stable reference genes is hence recommended for diurnal/circadian studies in plants.

## Introduction

Plants perceive their environment as a daily change in light and temperature conditions known as photocycles and thermocycles, respectively that vary with different seasons and regions (latitude) on the planet. These sessile organisms, like all animals, have evolved a 24 h period circadian clock that synchronizes their internal physiology and metabolism to the correct time of the day with the daily photo- and thermo-cycle. This is an important means of their assessment of the time of day and season^1–3^. Circadian clock benefits the plants with increased fitness and vigour and provides an adaptive advantage as they are able to anticipate daily and seasonal environmental variations and make appropriate changes in their metabolism^4,5^. It has been found that up to 89% of genes in *Arabidopsis*, when entrained in a day-night cycle, show daily cyclic changes (oscillate) in their transcript levels with a peak at some point of the day; such genes are diurnally controlled. About 10–30% genes are such that will continue to oscillate with the same rhythm, for some days, even when transferred to a free running or constant condition, i.e. when there is no day-night cycle, and such genes are called circadian genes^1,2^. These genes continue to drive the circadian rhythms of plants, at least for some days, even in the absence of external environmental cues. Nearly all the genes associated with hormone biosynthesis and signaling and the integrated stress signaling pathway are regulated by the circadian clock that synchronizes them for better growth and development and improved fitness^6–8^.

Study of the involvement of a gene in the functioning of the circadian clock oscillator, or if the gene is controlled by the circadian clock itself, frequently requires gene expression profiling to be carried out of the gene itself and its effect on the signature output pathway genes in diurnal as well as circadian conditions (free running). It becomes a mammoth task as the sample number is invariably high in studies dealing with circadian gene expression and the precision required is also high. Real-time quantitative PCR (RT-qPCR) has been widely accepted and employed as a method for gene expression quantification in plant science research^9^. It is the method of choice for high throughput, precise and speedy quantification of native genes in diurnally entrained and/or free running (circadian) tissue samples for studying cyclic expression patterns. The technique has become all the more powerful with the introduction of nano-litre plates that can analyze 3072 samples at the same time^10^ and the microfluidic technology where gene expression is measured using dynamic arrays, empowering the users to analyze 9216 samples in the same run^11^. Having said this, it would be appropriate to talk about the shortcomings and careful considerations that need to be addressed for a correct and meaningful analysis using RT-qPCR, as the technique is prone to both human and technical errors. After careful tissue sampling, RNA extraction and purification, and cDNA synthesis with all possible quality and quantity checks and minimized pipetting errors, the most important factor to be considered is the selection of one, or more appropriately, more than one most suitable house-keeping genes (also known as internal control or reference genes) for normalizing the data^12,13^. The most important criteria for selection of house-keeping genes for an experiment is that its expression level should remain stable across all the samples to be analysed^12,14–16^. These genes were initially thought to play central roles in house-keeping activities or basic cellular processes in the cell and hence were presumed to be constantly expressed and unregulated. Later, with the growing popularity of this technique, it was found that these house-keeping genes were also regulated under certain conditions^17,18^, hence their preference as control genes vary for different sample categories^19^. In general, there are no fixed internal control genes that can be used for all RT-qPCR analyses and the choice of these genes differ with experiment type to be performed, such as exposure to abiotic stresses, hormonal treatments, developmental stages, diurnal studies and other treatments. An internal control best suited for one experimental set may vary in another set of treated tissues and hence may be rendered unsuitable, which is the case for most of the known house-keeping genes in plants. Choosing an inappropriate internal control can lead to anonymous errors and eventually to serious misleading interpretations of results and conclusions^12–14,16,19^.

Rice is not only a major food crop in several Asian countries, feeding millions of people, but is also a model system for genetic and genomic studies in monocots as its genome is fully sequenced to finished quality and very well annotated. It is the simplest among all the major cereals, such as wheat and maize, and shows good genetic correlations, making the studies with other cereal crops easier. Over the past decade, several house-keeping genes have been identified across cereals, legumes, shrubs and woody perennials and evaluated for their stability under different conditions using freely available statistical algorithms, such as GeNORM^12,20^, BestKeeper^21^, NormFinder^22^, ∆Ct^23^, RefFinder^24^, etc. Many new house-keeping genes have been identified in *Arabidopsis*^25^ and in rice^26^ for a wide variety of sample types. These studies are sufficient to assess the possible best internal control genes for some common set of experiments across many other species but there is a continuous need for expanding the list of experiments and sample types. Until now, to the best of our knowledge, there has been no proper validation of house-keeping genes for diurnal and circadian studies in dicots or monocots.

In this study, we have attempted to evaluate 10 commonly used house-keeping genes, earlier validated for rice in our laboratory^27^, for their utility as best suitable internal controls for diurnal and circadian expression profiling in rice for normalizing RT-qPCR data and also reinforce the importance of using multiple genes as superior internal controls. Also, the reliability of the results was confirmed by normalizing the RT-qPCR data of 5 known circadian genes against the chosen 10 reference genes and analyzing their expression profiles. We have shown that the expression of some of the popular house-keeping genes in rice oscillates with a circadian rhythm and hence are unsuitable for normalizing RT-qPCR data in diurnal/circadian studies. Furthermore, we conclude that the geometric mean of three reference genes is best for normalizing circadian RT-qPCR data.

## Results

### Selection of candidate house-keeping genes, primer designing, amplification efficiency and specificity

**Figure 1:**
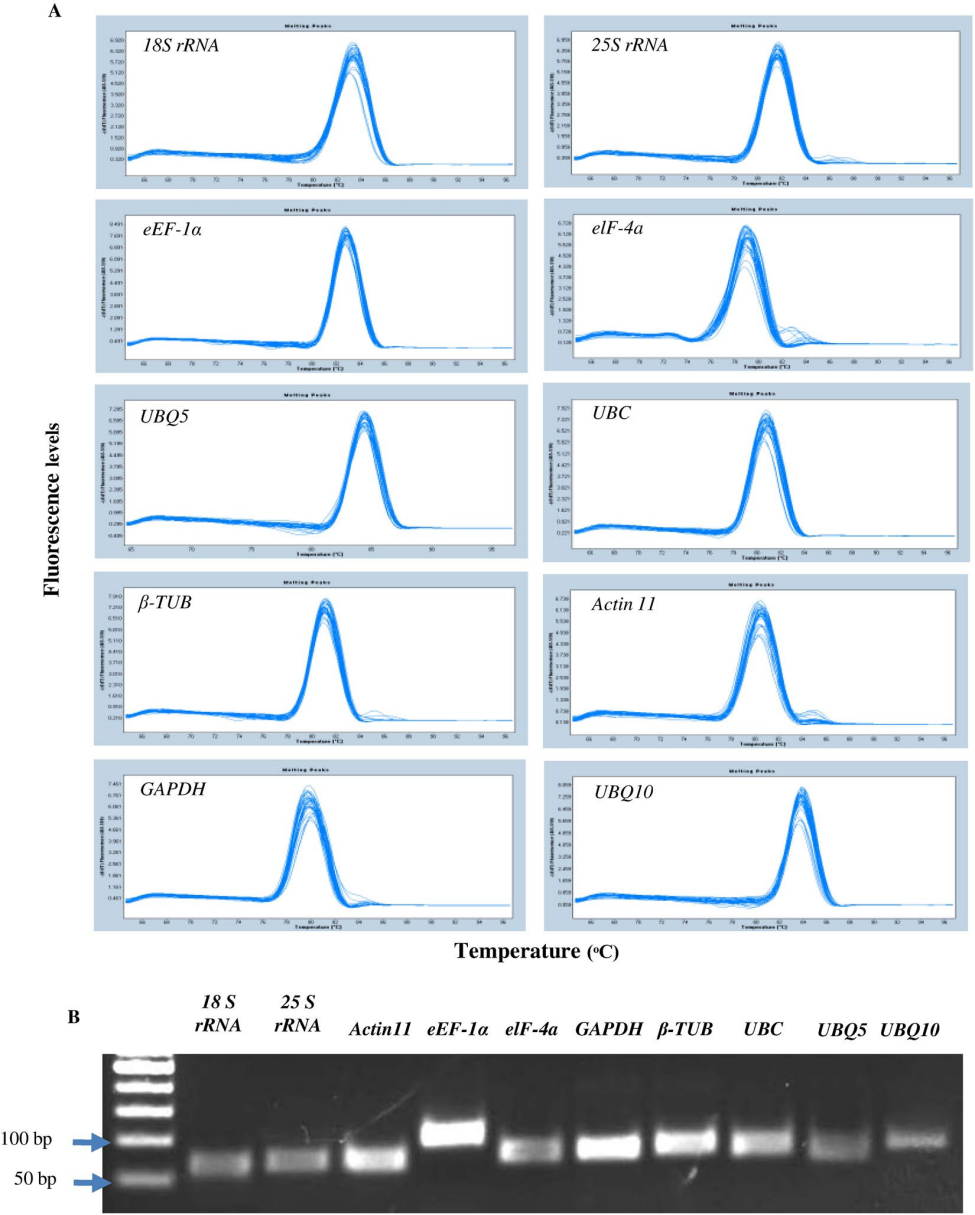
Specificity of the primers used in RT-qPCR amplification. (**A**) Melting curves of all the 10 house-keeping genes showing single peaks obtained from the three technical replicates of the first day samples (12 cDNA samples). (**B**) 2% agarose gel picture showing amplification of specific PCR products of expected sizes by the primer pairs of each of the house-keeping genes used in this study.

In this study, a total of 10 house-keeping genes from rice were considered for analysis based on a previously published study from our laboratory^27^. We had chosen some of the most commonly used house-keeping genes at that time, when the rice genome was sequenced and made publically available (IRGSP 2005)^28^ and was being actively annotated; our group also actively participated in this initiative. These genes are still very popular amongst the researchers across the globe and numerous studies have been carried out utilizing these genes for normalization of the RT-qPCR data. In fact, utility of these genes has been already demonstrated in a variety of plant tissues (developmental stages) as well as treated samples by many groups. The PCR efficiencies for all the primer pairs tested in our study ranged between 93% (*eEF-1α*) and 107% (*eIF-4a*) (Table 1). The melting curve analysis revealed single peaks for each primer pair indicating the specificity of the PCR reactions (Fig. 1A), which was also confirmed by analyzing the final PCR products on 2% agarose gel that showed presence of a single band of expected sizes (Fig. 1B). The primers, thus used, were efficient and specific enough for accepting them for routine RT-qPCR analysis.

**Table 1:** List of primers and amplification efficiencies of the house-keeping genes.

Gene Name^a^	Accession no.^b^	Gene Description	Primer Sequence^*c*^	Amplicon length (bp)	Primer efficiency	TIGR Locus ID
*eEF-1α*	AK061464	Eukaryotic elongation factor1-alpha	5′-TTTCACTCTTGGTGTGAAGCAGAT-3′ 5′-GACTTCCTTCACGATTTCATCGTAA-3′	103	93.7	LOC_Os03g08020
*UBC*	AK059694	Ubiquitin-conjugating enzyme E2	5′-CCGTTTGTAGAGCCATAATTGCA-3′ 5′-AGGTTGCCTGAGTCACAGTTAAGTG-3′	76	101	LOC_Os02g42314
*ACT11*	AK100267	Actin 11	5′-CAGCCACACTGTCCCCATCTA-3′ 5′-AGCAAGGTCGAGACGAAGGA-3′	67	99.5	LOC_Os03g50885
*GAPDH*	AK064960	Glyceraldehyde-3-phosphate dehydrogenase	5′-AAGCCAGCATCCTATGATCAGATT-3′ 5′-CGTAACCCAGAATACCCTTGAGTTT-3′	79	105.3	LOC_Os04g40950
*β-TUB*	AK072502	Beta-tubulin	5′-GCTGACCACACCTAGCTTTGG-3′ 5′-AGGGAACCTTAGGCAGCATGT-3′	82	98	LOC_Os01g59150
*eIF-4a*	AK073620	Eukaryotic initiation factor 4a	5′-TTGTGCTGGATGAAGCTGATG-3′ 5′-GGAAGGAGCTGGAAGATATCATAGA-3′	76	106.9	LOC_Os02g05330
*18S rRNA*	AK059783	18S ribosomal RNA	5′-CTACGTCCCTGCCCTTTGTACA-3′ 5′-ACACTTCACCGGACCATTCAA-3′	65	99.9	LOC_Os09g00999
*UBQ5*	AK061988	Ubiquitin 5/40 S ribosomal protein S27a	5′-ACCACTTCGACCGCCACTACT-3′ 5′-ACGCCTAAGCCTGCTGGTT-3′	69	106	LOC_Os01g22490
*25S rRNA*	AK119809	25S ribosomal RNA	5′-AAGGCCGAAGAGGAGAAAGGT-3′5′-CGTCCCTTAGGATCGGCTTAC-3′	68	97.1	LOC_Os09g01000
*UBQ10*	AK101547	Ubiquitin 10	5′-TGGTCAGTAATCAGCCAGTTTGG-3′ 5′-GCACCACAAATACTTGACGAACAG-3′	81	102.2	LOC_Os02g06640

^a^Gene name based on similarity to *Arabidopsis* proteins.

^b^Full length cDNA accession number.

^c^The upper line represents the forward primer sequence and the lower line represents the reverse primer sequence.

### Expression profiling of the house-keeping genes

**Figure 2:**
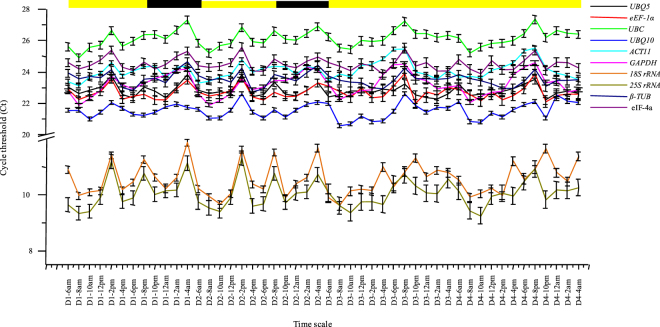
The mean Ct values of all the house-keeping genes. The graph shows the overall variation in the Ct values for each of the house-keeping genes across all the 48 samples. The Ct values of *18S rRNA* and *25S rRNA* were very low indicating extremely high expression levels. The error bars represent standard error. The average Ct values for all the house-keeping genes have been given in Supplemental Table S1.

**Figure 3:**
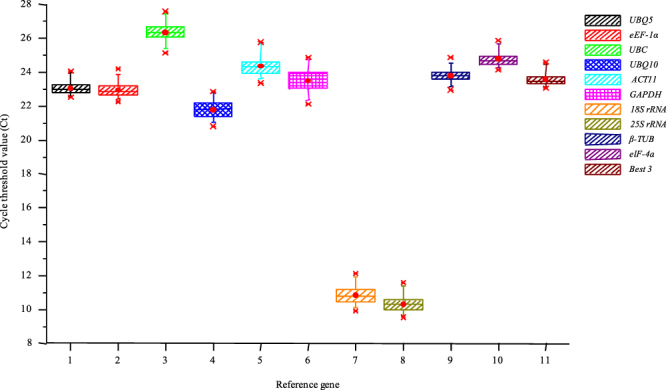
Variation in Ct values of the house-keeping genes across all the cDNA samples. Box plot graph showing variation in Ct values for each of the house-keeping genes across all the 48 diurnal/circadian samples. The median values are represented as lines across the box. The lower and the upper boxes represent the 25^th^ and 75^th^ percentile, respectively. Whiskers represent the maximum and minimum values.

The amplification plots of all the house-keeping genes are given in Supplemental Fig. S1 that shows efficient amplification rates for all the primer pairs. The mean of the three technical replicates of the two biological replicates (six Ct values in total) were averaged and used for all further analyses; these values have been presented in Supplemental Table S1. The mean Ct values for the 10 reference genes across the 48 diurnal and circadian samples ranged from 9.34 for *25S rRNA* to 27.34 for *UBC* (Fig. 2). While the Ct values for *UBQ5* (22.79 ± 0.39 SD, 1.71% CV), *UBQ10* (21.54 ± 0.49 SD, 2.3% CV), *eEF-1α* (22.67 ± 0.45 SD, 2% CV), *ACT11* (24.11 ± 0.56 SD, 2.34% CV), *GAPDH* (23.20 ± 0.70 SD, 3.02% CV), *β-TUB* (23.52 ± 0.42 SD, 1.8% CV), and *eIF-4a* (24.49 ± 0.42 SD, 1.74% CV) lied mostly between 20 and 25 and hence they were moderately expressed, the Ct values for *UBC* (26.11 ± 0.53 SD, 2.04% CV) were higher and ranged between 25 and 27, which makes it the lowest expressing, amongst the 10 genes selected for this study. The *18S rRNA* (10.56 ± 0.59 SD, 5.57% CV) and *25S rRNA* (10.05 ± 0.50 SD, 5.03% CV) transcripts were found to be extremely abundant and their Ct values ranged between 9 and 11. The coefficient of variation for all the genes ranged from 1.71%–5.57%. The expression variability across all the samples for all the house-keeping genes (Fig. 3) was higher for *GAPDH*, *ACT11*, *UBQ10*, *UBC*, *18S rRNA* and *25S rRNA* and lower for *UBQ5*, *eEF-1α*, *β-TUB* and *eIF-4a*. Moreover, this expression variability was least when the geometric mean of *UBQ5*, *eEF-1α* and *eIF-4a* (Ct 23.3 ± 0.39 SD, 1.69% CV) was taken for normalization.

### Expression stability analysis of the house-keeping genes

The mean Ct values were directly imported to the four algorithms used in this study, i.e. GeNORM, NormFinder, BestKeeper and ∆Ct that calculate the stability of the genes across the entire sample set in an experiment. In general, all the house-keeping genes appeared to be stable (Fig. 2) with overall less variation in their expression values but their suitability for diurnal and circadian studies needed further assessment and confirmation.

**Figure 4:**
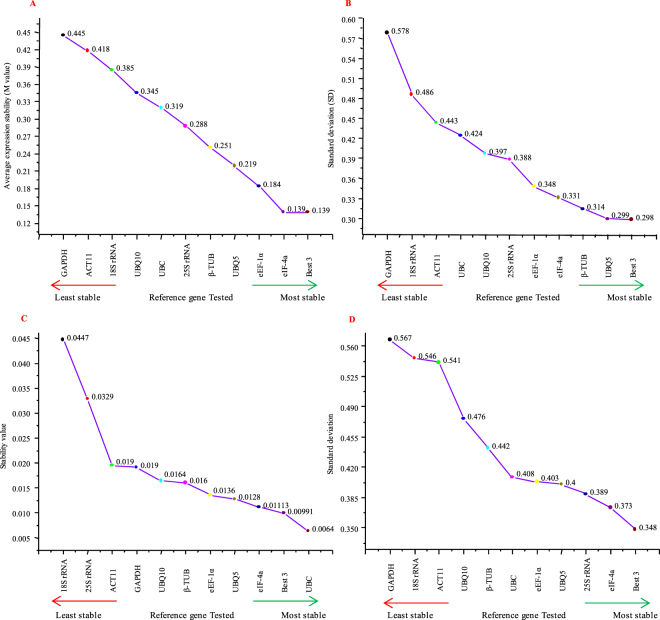
Expression stability analysis and ranking of the house-keeping genes by the four algorithms used in this study. Gene expression stability plots and subsequent ranking of the house-keeping genes by (**A**) GeNORM, based on average stability value (M), (**B**) BestKeeper, based on the standard deviation values, (**C**) NormFinder, based on the stability values calculated by the software, and (**D**) ∆Ct, based on the standard deviation values. The most stable genes are on the right hand side and the least stable genes are arranged on the left. The stability values, for each gene, by all the four programs are indicated on the curves.

GeNorm measures the stability of a reference gene based on the principle that the expression ratio between two ideal reference genes should remain the same under all experimental conditions and in the tissues being analyzed. Any deviation from this ratio will reflect either one or both reference genes being not constantly expressed and any increase in this ratio will mean a corresponding decrease in gene stability. The algorithm assigns a gene stability value M to all the reference genes, which is the average pairwise variation of a gene with all other reference genes included in the study. The genes with least M value have the most stable expression and stepwise exclusion of genes with highest M-values will result in a combination of two genes with highest stability^12^. *eIF-4a*, *eEF-1α* and *UBQ5* were predicted to be the most stable genes by GeNORM with least M-values of 0.139, 0.184 and 0.219, respectively (Fig. 4A). *GAPDH*, *ACT11* and *18S rRNA* were found to be least stable with the M-values of 0.445, 0.418 and 0.385, respectively. Nevertheless, all the genes analyzed showed good overall stability with their M values much lower than the set threshold (M < 1.5) by Vandesompele *et al*.^12^. The BestKeeper algorithm determines the best stable reference genes out of a set of at least 10 genes for an experimental set, based on the principle that the expression of good reference genes are highly correlated and constructs a correlative index out of it, which is then compared with the target genes to reach a conclusion whether or not the reference genes are regulated^21^. *UBQ5*, *β-TUB* and *eIF-4a* were predicted to be most stable by the BestKeeper algorithm with the stability values (standard deviation) of 0.299, 0.314 and 0.331, respectively, followed by *eEF-1α* (stability value 0.348). *GAPDH* (0.577), *18S rRNA* (0.487) and *ACT11* (0.443) were the least stable genes (Fig. 4B). The cut-off value for this stability measure is 1, below which all the genes are deemed to be stably expressed; this was the case for all the genes investigated in this study. NormFinder is based on the principle that any house-keeping gene will show some extent of variation across the entire set of samples to be studied in an experiment. Hence, to improve the stability measure of the reference genes, it takes into account this overall variation and, in addition, it also considers the variation between different subgroups of samples in the experiment, such as control and treated samples^22^. NormFinder predicted *UBC*, *eIF-4a* and *UBQ5* to be the most stable genes with stability values of 0.0064, 0.011 and 0.013, respectively, followed by *eEF-1α* (0.0135), whereas *18S rRNA*, *25S rRNA*, *ACT11*, and *GAPDH* were the least stable with stability values of 0.447, 0.329, 0.0195 and 0.0191, respectively (Fig. 4C). The ∆Ct approach compares the relative expression of pairs of genes for each sample in an experiment and ranks the reference genes for their stability according to repeatability of the expression difference across all the samples^23^. According to the ∆Ct approach, *eIF-4a* (SD = 0.373), *25S rRNA* (SD = 0.389) and *UBQ5* (SD = 0.4) were the most stable genes followed by *eEF-1α* (SD = 0.403), whereas *GAPDH* (SD = 0.567), *18S rRNA* (SD = 0.546) and *ACT11* (SD = 0.541) were the least stable (Fig. 4D). Taking together, the results suggest *eIF-4a*, *UBQ5* and *eEF-1α* to be the most stable genes as all four algorithms have given them high ranking for their stability. In comparison, *GAPDH*, *18S rRNA* and *ACT11* were given the least rankings by all the algorithms and hence were the least stable.

### Optimization of the number of reference genes required for RT-qPCR analysis

**Figure 5:**
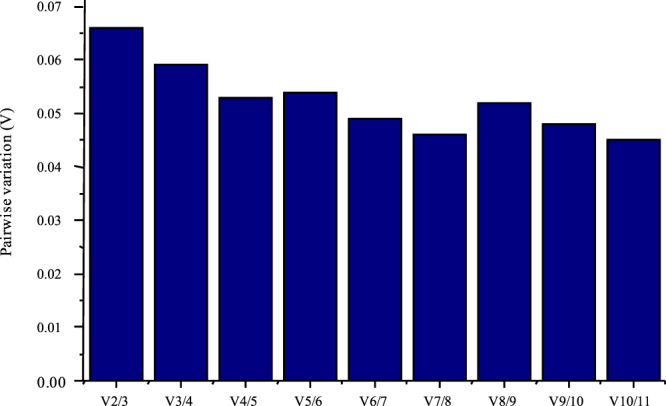
Pairwise variation (V) analysis of the house-keeping genes by GeNORM for finding out the optimal number of genes for data normalization. The pairwise variation (V_n_/V_n+1_) was analyzed between normalization factors NF_n_ and NF_n+1_. The default cut-off value was 0.15, below which all gene pairs were considered stable with no need to add the third gene for normalization.

It is also important to know the optimum number of reference genes that are required to normalize RT-qPCR data for the given samples in an experiment. Based on the stability of expression, GeNorm is also programmed to evaluate the number of house-keeping genes required to normalize RT-qPCR data. For this purpose, the three most stable genes (with least M-values) are used to calculate the normalization factor (NF_n_, n = 3) and stepwise inclusion of one more reference gene until the inclusion of (n + 1)^th^ gene has no significant effect on the newly arrived normalization factor (NF_n+1_). Pairwise variation, V_n/n+1,_ calculated between the sequential normalization factors, NF_n_ and NF_n+1_, if large, means that the added gene has a significant contribution for a reliable normalizing factor and should be included in the analysis. If this variation is negligible or low, it means the newly added gene has no significant contribution towards the normalizing factor and is virtually unnecessary^12^. Taking into consideration, the recommended pairwise variation cutoff value (V < 0.15), the geometric mean of two most stable reference genes, i.e. *eIF-4a*/*UBQ5*, *eIF-4a*/*eEF-1α* or *eEF-1α*/*UBQ5*, is sufficient to normalize diurnal/circadian RT-qPCR data in rice, and no third gene is required, as the pairwise variation was far less than the set cut-off (0.15) for all the genes (Fig. 5). Keeping in mind that certain genes show very weak cyclic patterns or have a very low transcript abundance, and can be badly affected by the reference gene selection, it is recommended that the geometric mean of three most stable reference genes (*eIF-4a/UBQ5/eEF-1α*) be taken for normalization of diurnal/circadian RT-qPCR data in rice. Although, there was no significant improvement in the stability value of geometric mean of the three genes (M = 0.139, GeNorm; SD = 0.298, BestKeeper; stability value = 0.001, NormFinder and SD = 0.348, ∆CT), it has been ranked number one by GeNorm, BestKeeper and ∆Ct methods and number two by NormFinder, making the combination most stable.

### Validation of the house-keeping genes for normalizing diurnal/circadian RT-qPCR data in rice

Firstly, the geometric mean of *eIF-4a*/*UBQ5*/*eEF-1α* was used to analyze the expression patterns of all the 10 reference genes. Secondly, in order to find out the effects of the individual house-keeping genes and the geometric means of the three most and least stable house-keeping genes on the expression patterns of known diurnal/circadian genes, the expression data of five known diurnal/circadian genes, viz. *OsLHY/CCA1*, *OsTOC1*, *OsFKF1*, *OsRVE1* and *OsRVE8*, were normalized and analyzed. Thirdly, the reference genes were interchangeably used to normalize the expression of the remaining reference genes to see their individual effects on the expression patterns of non-cyclic genes and genes with weak cyclic patterns.

**Figure 6:**
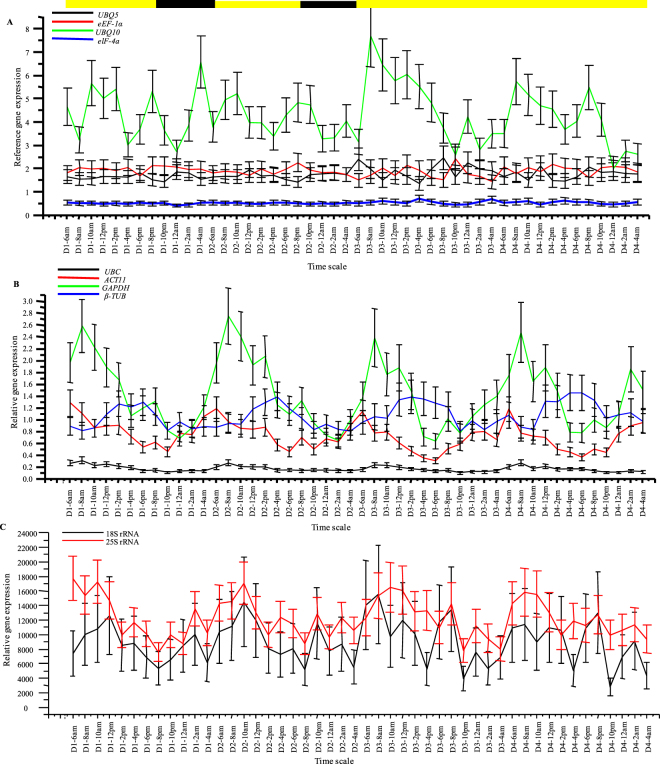
Expression profiles of all the house-keeping genes analysed in this study normalized with the geometric mean of the best three stable house-keeping genes. Relative expression profiles of all the house-keeping genes were obtained after normalization with the geometric mean of the three best stable genes, *eIF-4a*, *UBQ5* and *eEF-1α*, as determined by the four computer algorithms used in this study. The house-keeping genes have been categorized into three sub-groups; (**A**) genes that did not show cyclic expression patterns, viz. *eIF-4a*, *UBQ5*, *eEF-1α* and *UBQ10*; (**B**) genes that showed cyclic expression patterns, viz. *UBC*, *ACT 11* and *GAPDH*, that displayed morning peaked circadian patterns of expression, and *β-TUB*, that exhibited evening peaked circadian expression pattern; and finally (**C**) genes that showed extremely high expression levels, viz. *18S rRNA* and *25S rRNA*. The error bars represent standard error.

When the reference genes were studied for their expression profiles (Fig. 6), *eIF-4a*, *UBQ5* and *eEF-1α* showed no cyclic pattern when their expression was normalized with respect to the geometric mean of *eIF-4a*/*UBQ5*/*eEF-1α* and were very stable. The *UBQ10*, although did not show any cyclic pattern, was highly unstable and showed irregular peaks and troughs. *UBC* had a morning expression pattern with the peak forming two hours after dawn, ZT 2, in a circadian fashion, although the peaks were very shallow and the expression appeared stable. *ACT11* and *GAPDH* were clearly morning expressing genes, peaking immediately at dawn (ZT 0) and two hours after dawn (ZT 2), respectively. *GAPDH* showed sharp peaks whereas *ACT11* exhibited relatively shallow peaks and both the genes oscillated in a circadian fashion. *β-TUB*, on the other hand, showed evening expression pattern, also circadian in nature, and peaked at ZT 8–12, although the peaks were broad and shallow. *18S rRNA* and *25S rRNA* had expressions manifolds higher than the remaining reference genes and they even displayed morning cyclic patterns of expression (ZT 4), although *18S rRNA* appeared diurnal and *25S rRNA* cycled in a circadian fashion.

**Figure 7:**
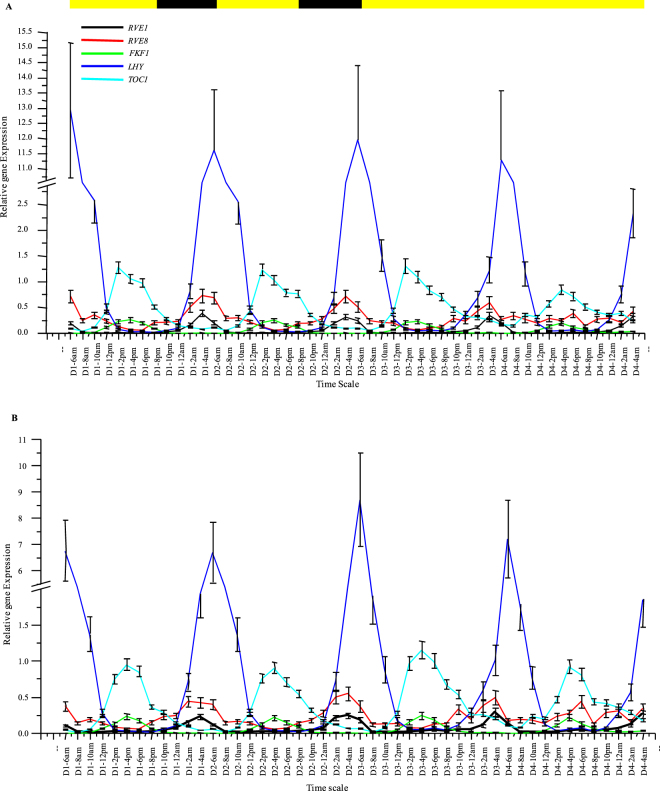
Expression profiling of the five circadian genes using the geometric means of the three most stable and three least stable house-keeping genes for normalization. The same cDNA samples for all the 48 time points, that were used to study the house-keeping genes, were used to generate the expression profiles of the 5 known circadian genes, viz. *LHY/CCA1*, *TOC1*, *FKF1*, *RVE1* and *RVE8*. The expression data of the circadian genes were normalized with the geometric mean of the three most stable house-keeping genes, (**A**) i.e. *eIF-4a*, *eEF-1α* and *UBQ5*, and the three least stable house-keeping genes, (**B**) i.e. *GAPDH*, *ACT11* and *UBQ10*, identified by the gene stability analyses.

The reference genes, when studied individually, had significant effect on the expression curves of the five known diurnal/circadian genes (Fig. S2). *OsLHY/CCA1*, *OsRVE1* and *OsRVE8* are morning expressing genes, forming sharp peaks at ZT 0, ZT 22 and ZT 22, respectively. *OsLHY/CCA1* and *OsRVE1* show clear circadian expression patterns whereas *OsRVE8* is a diurnal gene and its cycling gets disrupted in the free running conditions. *OsFKF1* and *OsTOC1* are evening expression circadian genes, showing peaks at ZT 8–10 and ZT 8–12, respectively. The cyclic expression profiles obtained were in accordance to the previously published microarray studies and are freely available in the public database www.ricearray.org (E-MEXP-2506 and E-MTAB-275). The minor differences, if any, may be due to small differences in the entrainment strategy. The *18S rRNA* and *25S rRNA* genes, due to their exceptionally high abundance levels diminished the cycling patterns of all the genes tested, including the robustly cycling *OsLHY/CCA1*, and thus should be grossly avoided for any diurnal/circadian RT-qPCR studies in rice. *UBQ10* also had considerably dwarfed the peaks of the test genes that could be attributed to its relatively higher expression levels. *UBC*, on the other hand, due to its lowest expression level among all the house-keeping genes, showed highly amplified cycling patterns of the test genes, but was still a good reference gene. Rest all the other house-keeping genes generated good cyclic expression patterns of the test genes with moderate peak amplitudes and somewhat similar diurnal/circadian patterns, i.e. broadening, narrowing or shifting the peaks by a couple of hours. Similar were the results when the geometric means of the three most stable genes, i.e. *eIF-4a*/*UBQ5*/*eEF-1α*, and the three least stable ones, i.e. *GAPDH*/*ACT11*/*UBQ10*, were used for normalization of the test genes (Fig. 7). There were only minor differences in the peak amplitudes or the cycling patterns of the test genes, when either of the two geometric means was used.

**Figure 8:**
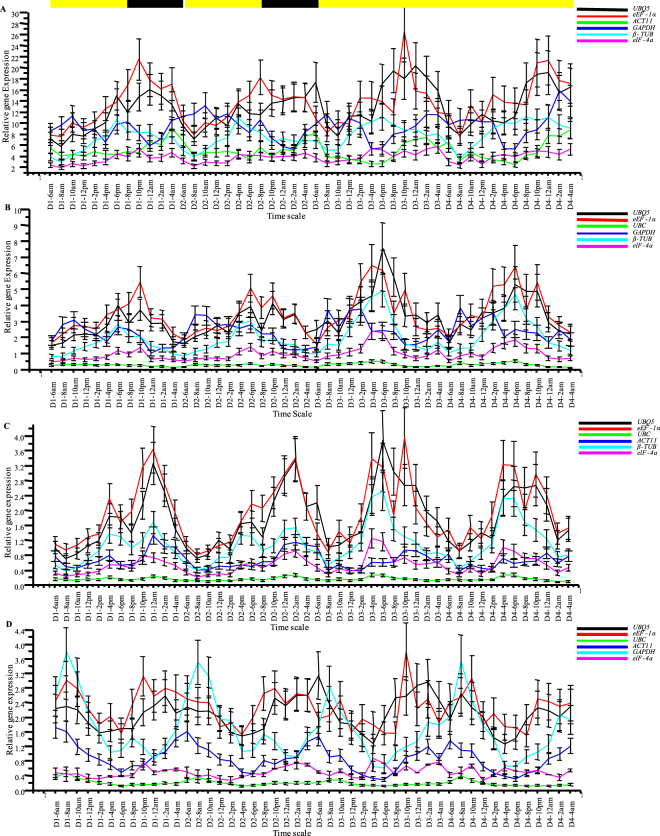
Effect of cyclic house-keeping genes on the expression profiles of non-cyclic genes and cyclic genes with weak cyclic expression patterns. The averaged Ct. values of the four cyclic house-keeping genes, i.e. the morning expressing genes *UBC* (**A**), *ACT11* (**B**) and *GAPDH* (**C**), and the evening expressing *β-TUB* (**D**) were used individually to normalize the relative expression data of rest of the house-keeping genes which were now treated as test genes. In all cases, the non-cyclic genes, viz. *eIF-4a*, *UBQ5* and *eEF-1α* developed cyclic patterns of expression when their relative expression profiles were normalized against the Ct. values of the cyclic house-keeping genes. The expression patterns of the cyclic genes, viz. *UBC*, *ACT 11*, *GAPDH* and *β-TUB* were greatly disturbed when their relative expression profiles were normalized interchangeably with each other. The error bars represent standard error.

The real test of these house-keeping genes was when dealing with test genes showing no cyclic patterns or weak cyclic patterns because the choice of house-keeping genes can dramatically affect their expression patterns (Fig. 8). For this purpose, the house-keeping genes showing cyclic patterns, i.e. *UBC*, *ACT11*, *GAPDH* and *β-TUB*, were interchangeably used to normalize expression of non-cyclic genes, *eIF-4a*, *UBQ5* and *eEF-1α*, and genes with weaker cyclic patterns, i.e. *UBC*, *ACT11*, *GAPDH* and *β-TUB*, all of them now treated as test genes. The morning expressing house-keeping genes, *UBC*, *ACT11* and *GAPDH*, when used individually to normalize the RT-qPCR data of the remaining house-keeping genes, now treated as test genes, converted the non-cyclic genes *eIF-4a*, *UBQ5* and *eEF-1α* to a cyclic pattern, circadian in nature, where they developed very broad but conspicuous expression peaks spanning mid-day, evening and mid-night and dropped sharply at dawn, the peak time for *UBC*, *ACT11* and *GAPDH*. The circadian rhythms of the morning expressing genes (*UBC*, *ACT11* and *GAPDH*) were disturbed dramatically as the peaks broadened, shifted or were completely diminished. The evening expressing gene *β-TUB* showed narrower evening peaks when normalized with *UBC* and *ACT11*, but developed an additional mid-night peak along with a narrower evening peak with *GAPDH*. When the evening expressing *β-TUB* was used for normalization, the non-cyclic *eIF-4a*, *UBQ5* and *eEF-1α* genes once again showed circadian patterns of expression with broad peaks spanning evening, midnight and morning time-points. Their peaks dropped in the mid-day to evening transition phase, the peak time for *β-TUB*. The morning circadian peaks of *UBC*, *ACT11* and *GAPDH* were not affected when normalized with *β-TUB* but their mid-day and evening expression levels were reduced making their morning peaks appear sharper and steeper.

## Discussion

The circadian clock controls the entire physiological and molecular functioning of plants, conferring them better adaptability in their ambient environment and enhanced vigor^4^. The clock functions by directing the expression of thousands of genes to peak at particular times in a 24-h day, when their products are needed for performing different physiological processes, thereby enabling better utilization of the plant’s energy and resources^1,2^. It is a routine practice to quantify gene expression levels throughout the day or even days together, when studying circadian clock functioning, which is an interplay of transcript abundance of thousands of genes in a 24 h period. RT-qPCR becomes the obvious choice for gene quantification in diurnal/circadian studies as it provides the opportunity of high-throughput and precise measurements in least possible time. Many genes have low expression levels and some genes show weak cyclic expression patterns, which tends to get easily maneuvered by selection of a not so suitable reference gene. This necessitates the need for standardization of some house-keeping genes for RT-qPCR analysis with diurnal/circadian samples, to facilitate reliable gene expression quantification. Several validation studies have been conducted in many plant species with different experimental samples where different house-keeping genes were deemed stable. Species to species variations are very common and, moreover, within a species, a gene which is stable for one set of samples may be unstable for another set of samples^19,29^. Keeping in mind that even the validated genes may show some variations across different samples, we began analyzing 10 house-keeping genes with a set of 48 diurnal and circadian samples, spread over four days. To the best of our knowledge, this work is the first of its kind detailed study of house-keeping genes for diurnal and circadian gene expression studies in plants. The genes chosen have already been studied previously with developmental, abiotic stress and hormonal treatment samples in rice by our group^27^. After confirming the primer efficiencies and specificities of the genes, the raw Ct values from the RT-qPCR data were averaged and analyzed for stability using four algorithms, viz. GeNORM, BestKeeper, NormFinder and ∆Ct. These four algorithms are based on different principles and independently calculate and rank the genes for their stability. *eIF-4a*, *eEF-1α* and *UBQ5* were predicted to be the most stable genes by GeNORM; *UBQ5*, *β-TUB*, *eIF-4a* and *eEF-1α* by BestKeeper; *UBC*, *eIF-4a*, *UBQ5* and *eEF-1α* according to NormFinder; and *eIF-4a*, *25S rRNA*, *UBQ5* and *eEF-1α* were the most stable as calculated by the ∆Ct method. On the other hand, *GAPDH*, *ACT11* and *18S rRNA* were the least stable according to GeNORM; *GAPDH*, *18S rRNA* and *ACT11* according to BestKeeper; *18S rRNA*, *25S rRNA*, *ACT11* and* GAPDH* according to NormFinder; and *GAPDH*, *18S rRNA* and *ACT11* were the least stable according to ∆Ct method. These results suggest that *eIF-4a*, *eEF-1α* and *UBQ5* are the most stable genes and *GAPDH*, *18S rRNA* and *ACT11* are the least stable, as predicted by all four programs. The minor variations observed in the gene rankings across all the four programs are obvious as they use different statistical approaches^30–33^. All the 10 genes, under study, showed good overall stability as their stability values were much below the default cut-off values set by the four programs. In the initial study by Jain *et al*.^27^ *eEF-1α* and *UBQ5* were found to be the most stable while carrying out RT-qPCR studies with rice samples of various developmental stages and abiotic stress and hormone treated tissues, and *UBQ10* was found to be the least stable, although *ACT11* and *β-TUB* were also ranked low in terms of stability, which supports our results. The only validation study, so far, for diurnal samples in lettuce by Sgamma *et al*.^34^ however, showed *GAPDH* to be relatively stable but *Actin* genes had relatively lower stability values. This may be partly due to different set of reference genes taken for analysis. *eEF-1α* and *eIF-4a* have been found to be very stable in samples representing various developmental stages and those subjected to diverse treatments in numerous plant species, such as switchgrass, *Brachiaria*, *Lolium*, perennial ryegrass, flax, potato, chickpea, cucumber, *Pyrus* etc^30,35–42^. *GAPDH* showed good stability in coffee, flax, sugarcane and pigeonpea for developmental tissues and certain stress treated samples^31,32,38,43,44^, but was expressed highly in some treated tissues in case of *Lolium*^36^. To make things all the more complicated *GAPDH* exhibited inter-cultivar differences of several folds with the same samples in rice and *Petunia*^45,46^. Gimeno *et al*.^30^ found *Actin* and *GAPDH* genes to be less stable under stress conditions in switchgrass. *Actin* genes were found to be unstable under salinity stress in potato, *Lolium* and cucumber^36,39,41^, but were considered stable under drought conditions in barley, only at specific developmental stages, thereby adding another dimension to the complexity in selecting reference genes^47^. The deviations observed in the otherwise stable expression levels of the reference genes, under certain conditions, may be due to some specific roles these genes might be playing apart from their main house-keeping function. For example, *GAPDH* is also involved in tRNA binding and age specific apoptosis in animal system^48,49^, which is not its usual role in glycolysis.

A general notion has developed over the past few years that the use of a single reference gene for normalization of an RT-qPCR data can introduce errors in the results and thus may be unreliable. The use of geometric mean of two to three best stable reference genes was therefore suggested by Vandesompele *et al*.^12^ for normalizing the data, which is getting much acceptance, worldwide. Hence, pairwise variation study by GeNORM was performed which predicted the geometric mean of only the two best genes to be sufficient as reference but we suggest the geometric mean of the best three genes, i.e. *eIF-4a*, *eEF-1α* and *UBQ5*, to be taken as reference for all diurnal/circadian studies in rice as it was concluded by all the four programs to be the most stable. Similar conclusions were given by Sgamma *et al*.^34^ where the geometric mean of the three best genes were recommended for diurnal samples in lettuce.

The reference genes showing the least stability should be grossly avoided as they tend to change the cyclic expression patterns of low expressing genes. In order to validate the stability results and to find out if these genes were good candidates for use in normalizing diurnal/circadian RT-qPCR data, the cycling patterns of five well known diurnal/circadian genes, viz. *OsLHY/CCA1*, *OsTOC1*, *OsFKF1*, *OsRVE1* and *OsRVE8*, were analyzed using, (1) each of the 10 reference genes alone and, (2) geometric mean of the 3 best stable and 3 least stable genes. The results indicated that all the reference genes were stable and did not cause any major shifts in the peaks of the test genes, viz. at ZT-0, ZT-22, ZT-22, ZT-8–10 and ZT-8–12 for *OsLHY/CCA1*, *OsRVE1*, *OsRVE8*, *OsFKF1* and *OsTOC1*, respectively, except for minor broadening of peaks, although the peak amplitudes were severely affected by the choice of the reference genes. The *18S rRNA* and *25S rRNA* transcripts levels were exceptionally high with the Ct values reaching in the order of 9–11 and almost completely diminished the peak expression levels of even the known test genes with sharp peaks, when used as reference. The *18S rRNA* and *25S rRNA* genes are therefore not recommended to be used for reference purpose with diurnal/circadian samples. Similar results were obtained by Jain *et al*.^27^ and Sinha *et al*.^31,32^. Their use as reference genes has been viewed with skepticism as its expression has been found to show variations, not only in different samples across different species, but also inter-cultivar variations were found^29^. Kim *et al*.^45^ however, found *18S rRNA* to be very stable for studying gene expression across various developmental stages in rice.

The geometric mean of the three most stable genes was used to study the expression patterns of the reference genes. *eIF-4a*, *UBQ5* and *eEF-1α* did not exhibit any cyclic patterns but, to our surprise, *GAPDH*, *ACT11* and *UBC* showed clear morning circadian rhythms whereas *β-TUB* showed evening circadian rhythms. A reference gene, which is itself undergoing cycling, cannot be used for normalizing diurnal/circadian RT-qPCR data. This became apparent when genes with no cyclic patterns or weak cyclic patterns were studied. When normalized with the morning peaked reference genes the non cyclic test genes developed midday to evening peaked cycling pattern and with evening peaked *β-TUB*, the test genes developed midnight to morning peaks. When the genes with weak cyclic patterns were studied with cyclic reference genes, the cycling patterns of the test genes were greatly distorted, to the extent of becoming arrhythmic in some cases. *GAPDH* and *ACT11* had scored badly in the stability rankings, but *UBC* and *β-TUB* were in the middle or high-up in those rankings, still none of the programs could detect the unsuitability of the four genes for diurnal/circadian studies. This reinforces the notion that the choice of house-keeping genes is experiment dependent and cannot be deemed best or universal for all sample types and plant species.

## Methods

### Plant material, day-night entrainment and sample harvesting

Rice seeds (*Oryza sativa* L. ssp. *Indica*, var. PB1) were obtained from the Indian Agricultural Research Institute, New Delhi. The seeds were dehusked and surface sterilized by washing with 70% ethanol for 1 min followed by two washes of 10 min each, with 0.1% HgCl_2_, 1% bavistin solution to which were added a few drops of teepol (detergent), with vigorous shaking. The seeds were subsequently rinsed, several times, with sterile reverse osmosis water and left overnight for soaking. Next day, seeds were blotted dry on sterile filter paper and inoculated on ½ MS salts supplemented with 1% sucrose and 0.4% phytagel (Sigma) for gelling, in magenta boxes. These boxes were transferred to a growth chamber (Conviron, Canada) under a daily photoperiodic cycle of 14 h light and 10 h dark. The temperatures were set at 35 °C in the light phase and 25 °C in the dark phase. After one week, the healthy seedlings were washed under tap water and transferred to liquid rice growth medium^50^ in culture tubes. After another two weeks in the same entrainment conditions, the healthy plants were harvested, in two biological replicates, every two hours for four consecutive days, beginning from the onset of the light period. During the third and the fourth day, the plants were given a free-running treatment of continuous light and hot condition, beginning the dawn of the third day. The harvesting of the plants was continued every two hours. The details of the light and temperature conditions and the sampling layout from the first to the fourth day have been summarized in supplemental Table S3.

### RNA isolation, quality checks and cDNA preparation

The total RNA was isolated independently from the two biological sets of samples, 48 samples in each set, using RNeasy Mini kit (Qiagen, USA) according to the manufacturer’s instructions. The isolated RNA was checked for its quality and quantity using Nanovue® spectrophotometer (GE Healthcare, UK). The samples had an A260/280 ratio between 1.9 and 2.1 and the A260/230 ratio between 2 and 2.3. The integrity of the RNA samples was also checked on a denaturing 1.5% agarose gel, which were then processed for cDNA synthesis. First-strand cDNA synthesis was done using the High Capacity cDNA Reverse Transcription kit (Applied Biosystems, USA) as per the manufacturer’s instructions, using 2 µg total RNA in a 40 µl reaction volume.

### Selection of the candidate house-keeping genes, primer designing and RT-qPCR analysis

In one of the first reference genes validation reports in rice by Jain *et al*.^27^ some of the commonly used genes were analyzed in various categories, such as abiotic stresses, hormonal treatments and developmental stages. In that study, some genes and gene pairs were established as good house-keeping gene candidates for all three categories. With the aim of extending that research for diurnal and circadian studies, the same genes and primer sets were used in this study also without any modification, details of which have been given in Table 1; see also Jain *et al*.^27^. The RT-qPCR analysis was carried out using 2 × Roche SYBR Green I master mix on a Roche Light Cycler 480 II instrument (Roche, Switzerland). The 18 µl reaction mix consisted of 6.25 µl PCR grade water, 9 µl 2 × Roche SYBR Green I master mix, 1 µl cDNA and 1.75 µl of 5 µM dilution of forward and reverse primers. Three technical replicates (5 µl each) from the above 18 µl reaction were subjected to the recommended thermal cycling profile of initial denaturation of 10 min at 95 °C and 45 cycles of 10 s at 95 °C, 20 s at 60 °C and 20 s at 72 °C. The expression values were obtained in the form of Ct (cycle threshold) values in RT-qPCR analysis, which is broadly defined as the cycle number at which the fluorescence levels in an amplification reaction clearly crosses the background noise. The fluorescence threshold levels were automatically determined by software of the RT-qPCR instrument. These Ct values were processed further mathematically to get the relative expression values for a gene. The completion of each run was followed by a melting curve analysis which denotes the specificity of the amplification. As the temperature rises from 60 °C to 95 °C, with an increment of 0.5 °C each time, the fluorescence levels are lost as the amplicons in the reaction continue to denature at increasing temperatures and the temperature at which half of the fluorescence is lost is denoted by Tm. This Tm is specific for each species of template in the reaction mix and the presence of more than one peak at the end of melting curve analysis shows non-specific amplifications. The amplification efficiencies in the RT-qPCR reactions for each primer pairs were calculated by making serial 10-fold dilutions of the pooled cDNA. The amplification efficiency is the rate of DNA amplification in any PCR reaction, which theoretically should be 100% implicating exponential doubling of the template DNA per PCR cycle. The final amplification products were also resolved on a 2% agarose gel to check for their specificity.

### Analysis of the house-keeping gene stability

For ranking the stability of the chosen house-keeping genes, the mean Ct (cycle threshold) values of the technical replicates from the two biological replicates were averaged, which were then directly imported to the four statistical algorithms, used in this study, that measures the stability value of the house-keeping genes, viz. GeNorm in the qBase Plus software version 2.5 (http://www.biogazelle.com/qbaseplus), NormFinder (http://www.moma.dk/normfinder-software), BestKeeper (http://www.gene-quantification.com/bestkeeper.html) and ∆Ct. The GeNorm programme also calculates the pairwise variation to identify the number of house-keeping genes that should be taken for analysis, for each experiment set.

### Validation of the reference gene stability

For validating the reference genes stability results, the expression profiles of 5 well known circadian genes, viz. *OsLHY/CCA1 (LOC_Os08g06110)*, *OsTOC1 (LOC_Os02g40510)*, *OsFKF1 (LOC_Os11g34460)*, *OsRVE1 (LOC_Os02g46030)* and *OsRVE8 (LOC_Os02g45670)* were investigated by performing RT-qPCR analysis of cDNA samples for all the 48 time points, in the same way as stated above. The expression values were normalized against each of the 10 selected reference genes, separately, as well as against the geometric mean of the three most stable and least stable reference genes. The primer details of these target circadian genes have been given in the Supplemental Table S2.

## Electronic supplementary material

Supplementary Information
