# Supplementary material for: Validation of house-keeping genes for normalization of gene expression data during diurnal/circadian studies in rice by RT-qPCR
Source: Sci Rep. 2018 Feb 16;8:3203. doi: 10.1038/s41598-018-21374-1 (PMC5816630; doi:10.1038/s41598-018-21374-1)
Supplement: Supplementary file 1 — Supplementary Information [file 41598_2018_21374_MOESM1_ESM.doc]

**Supplementary Information**

Validation of house-keeping genes for normalization of gene expression data during diurnal/circadian studies in rice by RT-qPCR

Nitin Jain, Satyam Vergish and Jitendra P. Khurana

**Supplementary Information**

**Supplementary Table S1: The mean Ct values of all the house-keeping genes across all the 48 samples.** The mean of the three technical replicates from the two biological replicates were averaged. These values were directly used as input for gene stability analysis.

| Sample name | *UBQ5* | *eEF-1α* | *UBC* | *UBQ10* | *ACT11* | *GAPDH* | *18S rRNA* | *25S rRNA* | *β-TUB* | *eIF-4a* |
| --- | --- | --- | --- | --- | --- | --- | --- | --- | --- | --- |
| D1-6am | 23.016667 | 22.88333 | 25.6067 | 21.535 | 23.39 | 22.77667 | 10.9067 | 9.64667 | 23.9167 | 24.60333 |
| D1-8am | 22.6 | 22.21 | 24.88 | 21.55 | 23.10667 | 21.88 | 9.96333 | 9.34 | 23.5367 | 24.18 |
| D1-10am | 22.816667 | 22.48333 | 25.5533 | 20.98 | 23.685 | 22.32667 | 10.09 | 9.41 | 23.69 | 24.38667 |
| D1-12pm | 23.006667 | 22.73333 | 25.6867 | 21.4267 | 23.91 | 22.83667 | 10.1367 | 9.91333 | 23.6167 | 24.77 |
| D1-2pm | 23.73 | 23.53 | 26.63 | 22.04 | 24.61 | 23.73 | 11.4233 | 11.1967 | 24.1233 | 25.40667 |
| D1-4pm | 22.545667 | 22.22667 | 25.62 | 21.6733 | 23.74333 | 23.17333 | 10.172 | 9.76333 | 22.9767 | 24.29 |
| D1-6pm | 22.313333 | 22.42667 | 25.95 | 21.3 | 24.06333 | 22.92333 | 10.448 | 9.89 | 22.8067 | 24.07 |
| D1-8pm | 23.02 | 22.54233 | 26.3467 | 21.2333 | 24.32 | 23.25333 | 11.26 | 10.7633 | 23.5133 | 24.62 |
| D1-10pm | 22.75 | 22.20333 | 26.3633 | 21.4267 | 24.38667 | 23.585 | 10.6233 | 10.0167 | 23.58 | 24.18 |
| D1-12am | 22.34 | 22.18667 | 26.08 | 21.78 | 23.61667 | 23.78 | 10.2033 | 10.1433 | 23.29 | 24.47 |
| D1-2am | 23.023333 | 22.91333 | 26.6667 | 21.95 | 24.306 | 24.1 | 10.61 | 10.1733 | 24.13 | 25.01 |
| D1-4am | 23.81 | 23.46333 | 27.29 | 21.7367 | 24.37 | 24.165 | 11.8733 | 11.1333 | 24.6267 | 25.32667 |
| D2-6am | 22.816667 | 22.68333 | 25.8067 | 21.625 | 23.3 | 22.57667 | 10.2067 | 9.74667 | 23.7367 | 24.38333 |
| D2-8am | 22.6 | 22.44 | 25.18 | 21.05 | 23.40667 | 21.9 | 9.92333 | 9.53 | 23.4467 | 24.28 |
| D2-10am | 22.716667 | 22.57333 | 25.6533 | 21.08 | 23.685 | 22.19667 | 9.65 | 9.41 | 23.57 | 24.34667 |
| D2-12pm | 22.606667 | 22.79333 | 25.7967 | 21.5767 | 23.82 | 22.62667 | 10.0367 | 9.91333 | 23.3267 | 24.57 |
| D2-2pm | 23.83 | 23.61 | 26.83 | 22.61 | 24.79 | 23.55 | 11.6233 | 11.3167 | 24.2233 | 25.60667 |
| D2-4pm | 22.406667 | 22.37667 | 25.9 | 21.4333 | 23.97333 | 22.87333 | 10.37 | 9.60333 | 22.7167 | 24.05 |
| D2-6pm | 22.533333 | 22.17667 | 25.82 | 21.06 | 24.25333 | 23.03333 | 10.19 | 9.68 | 22.9067 | 24.02 |
| D2-8pm | 23.33 | 22.67333 | 26.5967 | 21.5833 | 24.36 | 23.45333 | 11.52 | 10.7633 | 23.8133 | 24.82 |
| D2-10pm | 22.55 | 22.40333 | 26.0533 | 21.1167 | 24.33667 | 23.445 | 9.87333 | 9.71667 | 23.62 | 24.39 |
| D2-12am | 22.46 | 22.42667 | 26 | 21.58 | 23.85667 | 23.73 | 10.3833 | 10.0633 | 23.41 | 24.26 |
| D2-2am | 22.823333 | 22.80333 | 26.4267 | 21.95 | 24.35 | 24.3 | 10.61 | 10.1033 | 23.94 | 24.69 |
| D2-4am | 23.28 | 23.28333 | 26.89 | 22.0767 | 24.23 | 24.095 | 11.6733 | 10.7233 | 24.3967 | 24.95667 |
| D3-6am | 22.386667 | 23.06333 | 26.2333 | 21.985 | 23.46 | 23.21 | 9.88333 | 10.0733 | 23.7133 | 24.59667 |
| D3-8am | 22.526667 | 22.73333 | 25.525 | 20.5667 | 23.86667 | 22.265 | 9.60333 | 9.61667 | 23.44 | 24.36 |
| D3-10am | 22.763333 | 22.35 | 25.4267 | 20.6767 | 23.69333 | 22.55333 | 10.13 | 9.36667 | 23.33 | 24.06333 |
| D3-12pm | 22.763333 | 22.95 | 25.985 | 21.1833 | 24.41667 | 22.81333 | 10.1833 | 9.75333 | 23.2933 | 24.51667 |
| D3-2pm | 22.68 | 22.32667 | 25.9167 | 20.8333 | 24.5 | 22.86333 | 10.1367 | 9.75667 | 22.96 | 24.37667 |
| D3-4pm | 22.883333 | 22.39667 | 25.9833 | 20.8767 | 24.81667 | 23.83 | 10.9967 | 9.66 | 22.91 | 23.82667 |
| D3-6pm | 22.803333 | 23.10333 | 26.625 | 21.51 | 25.45 | 24.435 | 10.2833 | 10.38 | 23.4267 | 24.53 |
| D3-8pm | 23.206667 | 23.91 | 27.2133 | 22.6 | 25.45667 | 24.49 | 10.8067 | 10.72 | 24.2233 | 25.50333 |
| D3-10pm | 22.513333 | 21.95667 | 26.4133 | 21.86 | 23.99333 | 23.61 | 11.3167 | 10.32 | 23.5567 | 24.36667 |
| D3-12am | 22.34 | 22.70333 | 26.4133 | 21.4233 | 23.84333 | 23.43667 | 10.6333 | 10.0667 | 23.54 | 24.59 |
| D3-2am | 22.26 | 22.51 | 26.18 | 21.74 | 23.55333 | 22.92333 | 10.87 | 10.0433 | 23.5067 | 24.04 |
| D3-4am | 22.58 | 22.995 | 26.3033 | 21.7033 | 24.09 | 23.045 | 10.7833 | 10.5567 | 23.5633 | 24.04333 |
| D4-6am | 23.176667 | 22.86333 | 26.1333 | 22.1 | 23.671 | 23.12 | 10.5133 | 10.1233 | 23.8133 | 24.79667 |
| D4-8am | 22.476667 | 22.53333 | 25.185 | 20.8417 | 23.72667 | 22.065 | 9.90333 | 9.42667 | 23.56 | 24.17 |
| D4-10am | 22.563333 | 22.13 | 25.5867 | 20.7867 | 23.61333 | 22.44333 | 10.04 | 9.24667 | 23.42 | 23.86333 |
| D4-12pm | 22.533333 | 22.71 | 25.795 | 21.3833 | 24.11667 | 22.71333 | 10.2133 | 9.95333 | 23.2233 | 24.71667 |
| D4-2pm | 22.74 | 22.19667 | 25.8467 | 21.1333 | 24.3 | 22.76333 | 9.96667 | 10.0567 | 22.94 | 24.12667 |
| D4-4pm | 22.923333 | 22.46667 | 25.9833 | 21.5967 | 24.59667 | 23.83 | 11.1967 | 9.96 | 22.94 | 24.13667 |
| D4-6pm | 23.203333 | 22.93333 | 26.425 | 21.91 | 25.34 | 24.285 | 10.5333 | 10.48 | 23.3867 | 24.73 |
| D4-8pm | 23.506667 | 23.88 | 27.3433 | 22.1 | 25.52667 | 24.569 | 10.9107 | 10.932 | 24.1433 | 25.37333 |
| D4-10pm | 22.213333 | 22.06667 | 26.1733 | 21.06 | 24.23333 | 23.31 | 11.6517 | 9.83 | 23.0667 | 24.11667 |
| D4-12am | 22.63 | 22.47333 | 26.6133 | 22.5233 | 23.92333 | 23.41667 | 10.7933 | 10.1767 | 23.43 | 24.62 |
| D4-2am | 22.76 | 22.57 | 26.44 | 22.14 | 23.76033 | 22.72333 | 10.47 | 10.1483 | 23.4467 | 24.59 |
| D4-4am | 22.61 | 22.555 | 26.3833 | 22.0433 | 23.51 | 22.855 | 11.3733 | 10.2567 | 23.4833 | 24.24333 |

**Supplementary Table S2:** Primer sequences of the five circadian genes of rice used in this study for validation of the house-keeping genes.

| Gene Name^a^ | TIGR Locus ID | Gene Description | Primer Sequence |
| --- | --- | --- | --- |
| *OsFKF1* | LOC_Os11g34460 | *FLAVIN-BINDING, KELCH REPEAT, F BOX 1* | 5’- ACGTCACTGTGAGGCTTGAGATGAG -3’  5’- ACACGACCTCCAACTGTAAACTTCC -3’ |
| *OsLHY/CCA1* | LOC_Os08g06110 | *LATE ELONGATED HYPOCOTYL/ CIRCADIAN CLOCK ASSOCIATED 1* | 5’- AGGTTCCTTGAAGCCTTGAAACT -3’  5’- TCTTTGTCCCAACATGCTCTTCT -3’ |
| *OsTOC1* | LOC_Os02g40510 | *TIMING OF CAB EXPRESSION 1* | 5’- GCAGCCGAGTACCTGGTCAA -3’  5’- ACACATGGGTCCAGAGGTTCA -3’ |
| *OsRVE1* | LOC_Os02g46030 | *REVEILLE 1* | 5’- GCGGCGGAGAGCGAGCTGCTGCGATC -3’  5’- GTACTGTGGCGTGACGTGTCCGTGCG -3’ |
| *OsRVE8* | LOC_Os02g45670 | *REVEILLE 8* | 5’- TGTGAGGTCTCTTCCCTGGCCAAACTGG -3’  5’- GATACAATGTTGCAAAGTGCCTTCCTAGTGC -3’ |

^a^ Gene name based on similarity to *Arabidopsis* proteins.

**Supplementary Table S3:** Description of all 48 samples of the rice plants used in this study with details of temperature and light conditions during all the four days of harvesting for diurnal and circadian profiling of genes.

|  |  |  |  |
| --- | --- | --- | --- |
| Sample name | Sample Description | Light | Temperature (ᵒC) |
| S1 | Day1-6am | OFF-ON | 25-35 |
| S2 | Day1-8am | ON | 35 |
| S3 | Day1-10 am | ON | 35 |
| S4 | Day1-12 pm | ON | 35 |
| S5 | Day1-2 pm | ON | 35 |
| S6 | Day1-4 pm | ON | 35 |
| S7 | Day1-6 pm | ON | 35 |
| S8 | Day1-8 pm | ON-OFF | 35-25 |
| S9 | Day1-10 pm | OFF | 25 |
| S10 | Day1-12 am | OFF | 25 |
| S11 | Day1-2 am | OFF | 25 |
| S12 | Day1-4 am | OFF | 25 |
| S13 | Day2-6am | OFF-ON | 25-35 |
| S14 | Day2-8am | ON | 35 |
| S15 | Day2-10 am | ON | 35 |
| S16 | Day2-12 pm | ON | 35 |
| S17 | Day2-2 pm | ON | 35 |
| S18 | Day2-4 pm | ON | 35 |
| S19 | Day2-6 pm | ON | 35 |
| S20 | Day2-8 pm | ON-OFF | 35-25 |
| S21 | Day2-10 pm | OFF | 25 |
| S22 | Day2-12 am | OFF | 25 |
| S23 | Day2-2 am | OFF | 25 |
| S24 | Day2-4 am | OFF | 25 |
| S25 | Day3-6am | OFF-ON | 25-35 |
| S26 | Day3-8am | ON | 35 |
| S27 | Day3-10 am | ON | 35 |
| S28 | Day3-12 pm | ON | 35 |
| S29 | Day3-2 pm | ON | 35 |
| S30 | Day3-4 pm | ON | 35 |
| S31 | Day3-6 pm | ON | 35 |
| S32 | Day3-8 pm | ON | 35 |
| S33 | Day3-10 pm | ON | 35 |
| S34 | Day3-12 am | ON | 35 |
| S35 | Day3-2 am | ON | 35 |
| S36 | Day3-4 am | ON | 35 |
| S37 | Day4-6am | ON | 35 |
| S38 | Day4-8am | ON | 35 |
| S39 | Day4-10 am | ON | 35 |
| S40 | Day4-12 pm | ON | 35 |
| S41 | Day4-2 pm | ON | 35 |
| S42 | Day4-4 pm | ON | 35 |
| S43 | Day4-6 pm | ON | 35 |
| S44 | Day4-8 pm | ON | 35 |
| S45 | Day4-10 pm | ON | 35 |
| S46 | Day4-12 am | ON | 35 |
| S47 | Day4-2 am | ON | 35 |
| S48 | Day4-4 am | ON | 35 |


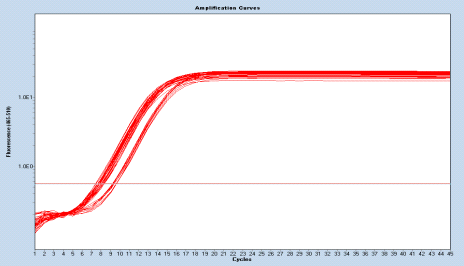

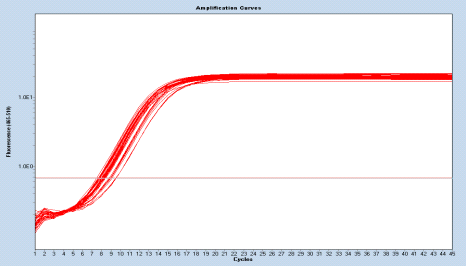

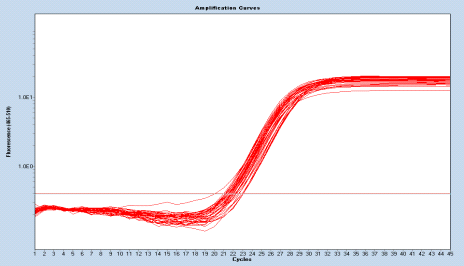

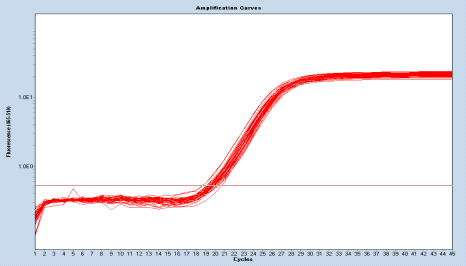

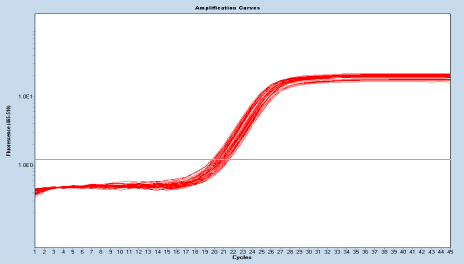

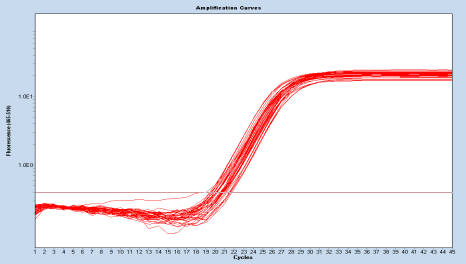

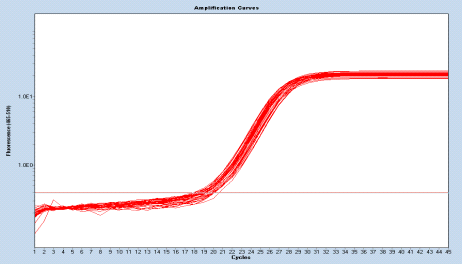

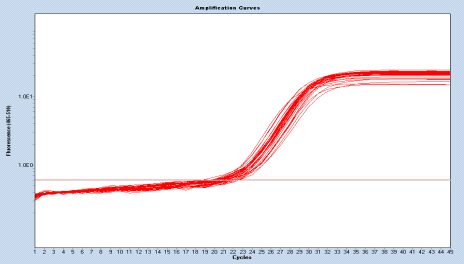

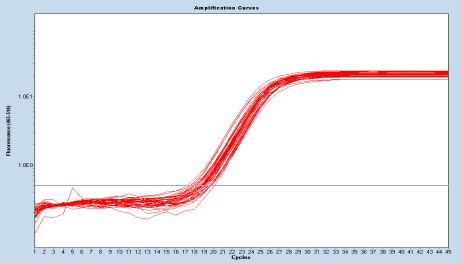

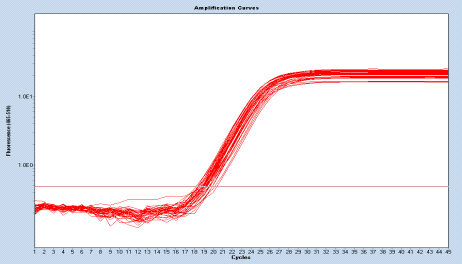


*18S*

*rRNA*

*25S*

*rRNA*

*Actin11*

*eEF*

*-*

*1α*

*elF*

*-*

*4a*

*GAPDH*

*β*

*-*

*TUB*

*UBC*

*UBQ5*

*UBQ10*

**Fluorescence levels**

**Number of cycles**

**Supplementary fig. S1: PCR amplification curves for all the house-keeping genes evaluated in this study.** The horizontal lines indicate the noise-cut-off level.

**Supplementary fig. S2: Effect of the individual house-keeping genes on the cyclic expression profiles of known circadian genes.** The same cDNA samples for all the 48 time points, that were used to study the house-keeping genes, were used to generate the expression profiles of the 5 known circadian genes viz. *RVE1*, *RVE8*, *LHY/CCA1*, *FKF1* and *TOC1*. Each of the 10 house-keeping genes was used individually to normalize the expression data of the circadian genes. The peak timings of the genes were not affected much but their abundance was severely affected. The gene curves when normalized with 18S RRNA and 25S RRNA are not visible in the graphs as the amplitudes of expression have been completely diminished due to very high expression levels of the two genes.
